# Age- and sex-standardised prevalence rates of fatigue in a large hospital-based sample of cancer patients

**DOI:** 10.1038/bjc.2011.251

**Published:** 2011-07-12

**Authors:** S Singer, S Kuhnt, R Zwerenz, K Eckert, D Hofmeister, A Dietz, J Giesinger, J Hauss, K Papsdorf, S Briest, A Brown

**Affiliations:** 1 Department of Health Psychology and Applied Diagnostics, University of Wuppertal, Wuppertal, Germany; 2 Department of Medical Psychology and Medical Sociology, University of Leipzig, Leipzig, Germany; 3 Department of Psychosomatic Medicine and Psychotherapy, University Medical Center, University of Mainz, Mainz, Germany; 4 Department of Rehabilitation Sports, University of Leipzig, Leipzig, Germany; 5 Department of Laryngo-Rhino-Otology, University of Leipzig, Leipzig, Germany; 6 Department of Psychiatry and Psychotherapy, Innsbruck Medical University, Innsbruck, Austria; 7 Department of Surgery, University of Leipzig, Leipzig, Germany; 8 Department of Radiation-Oncology, University of Leipzig, Leipzig, Germany; 9 Department of Obstetrics and Gynaecology, University of Leipzig, Leipzig, Germany; 10 Cancer Epidemiology Unit, University of Oxford, Oxford, UK

**Keywords:** fatigue, neoplasms, prevalence studies, prospective studies, mental health

## Abstract

**Background:**

The aim of this longitudinal study was to determine age- and sex-standardised prevalence rates of cancer-related fatigue in different groups of patients.

**Methods:**

This was a prospective study in a cohort of *N*=1494 cancer patients investigating fatigue at three time points t1–t3 (t1: admission to hospital, t2: discharge, t3: half a year after t1). Fatigue was measured with the Multidimensional Fatigue Inventory. Age- and sex-adjusted norms were derived from a representative community sample of *N*=2037, using a cutoff at the 75th percentile.

**Results:**

At admission to the hospital, 32% of the patients were classified as fatigued. At discharge, the overall prevalence rate was 40%, and at half a year after t1, prevalence was 34%. Fatigue prevalence rates differed according to tumour stage, site, age, and sex of the patients.

**Conclusion:**

The prevalence rates provided by this study can be used for the planning of research and clinical routine.

Fatigue is one of the major problems in cancer patients (Curt *et al*, 2000; Teunissen *et al*, 2007; Bruera and Yennurajalingam, 2010). Pain, the main complaint in previous times, has become less prevalent because of improved symptom management (Vogelzang *et al*, 1997); fatigue, however, remains a therapeutic challenge (Lower *et al*, 2009; Barsevick *et al*, 2010; Jean-Pierre *et al*, 2010; Moraska *et al*, 2010).

Unfortunately, fatigue often goes undetected by health-care providers, and physicians tend to underestimate its prevalence (Vogelzang *et al*, 1997). To be able to plan treatment and to conduct research studies, we need to know how widespread fatigue is among patients. Several prevalence rates have been published, especially in samples of patients undergoing treatment for breast or prostate cancer (Andrykowski and Jacobsen, 2010; Kyrdalen *et al*, 2010) as well as in survivors (Kuhnt *et al*, 2009). However, there is little knowledge regarding the patterns of fatigue over the course of treatment or among patients with rare cancers.

Therefore, with this prospective study, we aimed at investigating the prevalence rates of fatigue in a large sample of patients with heterogeneous types of tumour diseases over time.

To date, we lack an internationally accepted ‘gold standard’ or even an agreed list of criteria to determine whether a patient should be considered ‘fatigued’ or not. Instead, fatigue is usually conceptualised as a dimensional construct, assessed by questionnaires, where a necessarily arbitrary threshold is used to classify patients. One of the most frequently used questionnaires is the Multidimensional Fatigue Inventory (MFI) developed by Smets *et al* (1995), where thresholds can be set, for example, at 16 points (Hagelin *et al*, 2009) or at different points according to the age and the gender of the patients, as suggested by Schwarz *et al* (2003). The latter method allows controlling for the effect that women and older people generally experience more fatigue than men and younger persons. Thus, prevalence rates that have been standardised by age and sex are not confounded by age or sex effects.

Research questions of this paper were the following:

1. How many cancer patients are classified as fatigued at three time points: the beginning of inpatient treatment; at discharge; and half a year after diagnosis?
2. How much do prevalence rates differ according to age, sex, and tumour stage?

## Materials and methods

### Study design

We conducted a prospective cohort study with consecutive patient enrolment in a large university hospital in Leipzig, Germany. Each patient who was admitted to the Departments of Gynaecology, Urology, Surgery, and Radiation-Oncology for diagnosis or treatment of cancer from June 2002 to December 2004 inclusive was contacted. Additional eligibility criteria for patient recruitment were aged 18 years or older, having sufficient command of German, being in a physical and mental condition that allowed the patient to complete the questionnaires. Whether a patient was ineligible due to his or her condition was discussed by physicians and investigators on a case-by-case basis.

This study was approved by the institutional review board of Leipzig University and was performed in accordance with the ethical standards laid down in the 1964 Declaration of Helsinki.

### Assessment instrument

Fatigue was assessed using the MFI, an internationally validated, multidimensional self-administered instrument (Smets *et al*, 1995, 1996). The 20 items of this measure can be summarised by the following subscales: General Fatigue, Physical Fatigue, Mental Fatigue, Reduced Motivation, and Reduced Activity. Each subscale contains four items (scores range from 4 to 20, with higher scores indicating increased fatigue). General Fatigue was used to calculate the prevalence of fatigue (Holzner *et al*, 2002).

Socio-demographic and medical data, including disease and treatment-related history, were collected from medical records and from a questionnaire completed by the patients.

### Procedure

On the first or second day of inpatient oncological treatment, eligible patients were approached by a research assistant who explained the study and sought their consent to participate. After written informed consent had been given, patients completed the MFI for the first time (t1). The second assessment was made before the patient was discharged from the hospital (t2). Usually, the patients completed the questionnaires on their own and the research assistant collected the forms afterwards. If the patient requested help because of reading difficulties etc., support was offered at the time of collection. The median time between t1 and t2 was 13 days (range 1–164 days).

Six months after t1, the MFI was sent out by post to the patients together with a stamped envelope and the patients were asked to return their completed forms back within 2 weeks (t3). Non-responding patients were contacted by phone and were again asked to complete the questionnaire. The cutoff for accepting replies at t3 was 10 months after t1. The median time between t1 and t3 was 6.2 months (range 4.7–10.0 months, interquartile range 0.5).

### Analysis

Prevalence of fatigue was defined as follows: a patient was considered as being fatigued if his or her fatigue score was higher than that of 75% of people in the general population in the same gender and age group, that is, the thresholds were the age- and gender-adjusted 75th percentiles from a German representative community sample with *N*=2037 (Schwarz *et al*, 2003). These thresholds were calculated from the raw data and are displayed in Table 1. Note that the subjects in that community sample were not generally healthy as they were selected at random.

In addition, descriptive statistics (mean and s.d.) of all MFI subscales were computed for the three assessment points. Group differences were tested by binary logistic regressions. All statistical analyses were performed using STATA, version 11 (StataCorp., 2009).

## Results

### Sample characteristics

During the study period, 2913 patients were referred to the participating clinics. In all, 590 of them were not eligible for this survey, leaving 2323 patients who were contacted. In all, 1803 of these consented to take part, but in 253 cases, no measurement at t1 was possible due to organisational problems (e.g., the patient was not available because of diagnostics or treatment). Thus, 1550 patients were enrolled into the study at t1. Of these, 1494 patients completed an MFI questionnaire at the time of admission to the hospital (t1), 884 at discharge (t2), and 1035 half a year later (t3). Between enrolment and discharge (t2), 95 patients died. Another 15 died during the period from t2 to t3.

Reasons given for refusing to participate were diverse, for example: distrust in data protection (*n*=19); refusal to participate in any study (*n*=43); preferred not to speak about cancer (*n*=7); questions were considered too intrusive (*n*=27); no motivation (*n*=73); family does not wish that the patient participates (*n*=10); or the wish to rest (*n*=54). However, the majority of reasons given could not be categorised into any summary group or the patient refused to indicate a reason for decline.

Of the participating patients, 891 (59.6%) were males and 603 (40.4%) were females. The mean age was 60.2 years (s.d. 12.2); the youngest participant was 20 and the oldest was 92 years old.

In all, 810 patients presenting to the hospital were treated with surgery, 264 with radiotherapy, 221 with radio-chemotherapy, 48 with chemotherapy alone, 101 came for diagnostic procedures, and 50 for other procedures such as pain medication. Further socio-demographic and medical characteristics are displayed in Table 2.

### Prevalence of fatigue in total sample

At t1, 32% of the patients were classified as fatigued (mean score of the General Fatigue scale 10.3, s.d. 4.2). At discharge, the overall prevalence rate was 40% (mean score 10.9, s.d. 4.2); at t3, prevalence was 34% (mean score 10.8, s.d. 4.3).

Means and s.d. measured on the different subscales of the MFI can be found in Table 3. Note that the means represent raw (unadjusted) data whereas prevalence rates are adjusted for age and gender effects.

### Prevalence of fatigue in subsamples

Diverse rates of fatigue were seen when the sample was stratified by age group (see Table 4). More younger patients experienced fatigue than older patients using the age- and gender-adjusted norms. At admission, 53% of the participants <40 years old were classified as fatigued, compared with 22% of patients older than 60 years (OR=4.0, *P*<0.001), that is the odds of being classified as fatigued was four times higher in the younger age group than in the older age group. Before discharge from the hospital, 55% of the younger and 29% of the older patients reported increased fatigue (OR=3.2, *P*<0.001). Half a year after t1, prevalence rates were 44% in the younger and 23% in the older patients (OR=3.0, *P*<0.001).

Men were less likely to be classified as fatigued than women. At the time of admission to the hospital (t1), the prevalence of fatigue was 28% among men and 39% among women (OR=1.6, *P*<0.001). Before discharge (t2), prevalence was 33% and 47%, respectively (OR=2.0, *P*<0.001), and at t3, 31% and 38%, respectively (OR=1.5, *P*=0.008).

Patients with advanced tumours (UICC=IV) reported fatigue more frequently (up to 51% at t2) than patients with less advanced cancer; however, fatigue prevalence did not increase with increasing tumour stage. Patients with UICC III, for example, reported fatigue less commonly than patients with UICC I during the entire stay at the hospital and at half a year later (see Table 4 for details).

Regarding treatment subgroups, the second measurement point (at discharge, t2) shows that patients who received chemotherapy between t1 and t2 are most often affected with 66% of them classified as fatigued, whereas only 36% of patients after surgery are fatigued (OR=3.4, *P*=0.001). Other treatment regimes resulted in prevalence rates of 41–44% (OR=1.3, *P*=0.22 for radiotherapy *vs* surgery and OR=1.4, *P*=0.09 for chemoradiation *vs* surgery). Half a year after t1, fatigue prevalence decreased more or less in all treatment groups (see Table 4); however, changes after radiotherapy were only minor. If the treatment received between t1 and t3 is taken into consideration, the prevalence rates are similar; however, the differences are less pronounced (Table 5). Patients on and off treatment half a year after t1 report similar levels of fatigue (OR=1.5, *P*=0.35).

**Figure 1:**
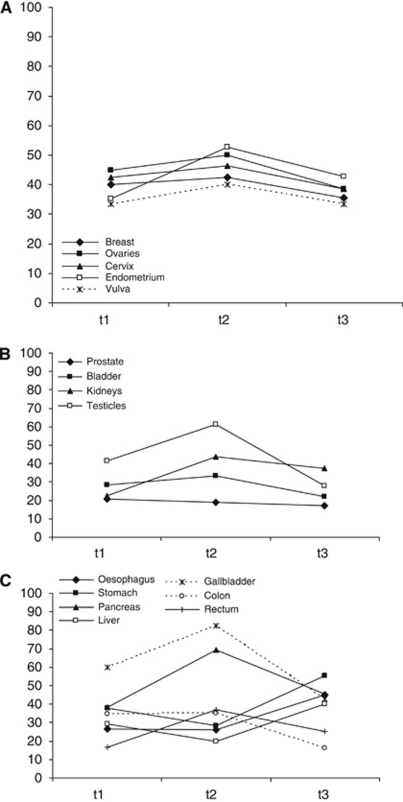
Fatigue prevalence over time in different groups of patients: (**A**) gynaecological malignancies, (**B**) urological malignancies, and (**C**) gastro-intestinal tumours.

Prevalence rates according to tumour entity are documented in Table 6 and Figure 1; rates are presented for only those tumour sites where, at each assessment point, data were available from at least five patients, to protect confidentiality. Although groups with *n*=5 are still small and estimation of prevalence is therefore imprecise, we think that presentation of these data are interesting and worthwhile reporting. To be able to evaluate the precision of the estimation, the 95% confidence intervals of each prevalence rate are displayed.

Highest prevalence rates were observed in patients with gall bladder cancer, but also in patients with head and neck, pancreatic, gynaecological, and haematological malignancies fatigue was frequent (for details see Table 6). The lowest rates with all three assessment points taken together (average of t1, t2, and t3) were seen in prostate cancer patients.

## Discussion

The aim of this investigation was to determine the prevalence rates of fatigue in different groups of cancer patients at the beginning of inpatient treatment, at the time of discharge, and half a year later. Based on our results, we can conclude that about a third of the patients at the time of admission to the hospital are suffering from fatigue. At discharge, the overall prevalence rate increases slightly to 40%, and half a year later, the prevalence is again one third. In different groups of patients, fatigue prevalence rates differ remarkably.

Although it is known that fatigue levels are in part predicted by medical, socio-demographic, and psychological factors, prevalence rates have not been published regularly to date. The main reason for that may be that fatigue is usually assessed with dimensional instruments and no consensus exists regarding which questionnaire should be used. Although the MFI can be viewed as one of the most widely accepted measures for use with cancer patients (Meek *et al*, 2000; Brix *et al*, 2009; Heutte *et al*, 2009; Agasi-Idenburg *et al*, 2010; Purcell *et al*, 2010; Riesenberg and Lubbe, 2010), the originators of the MFI (Smets *et al*, 1995, 1996) did not determine a cutoff point at which fatigue could be considered as ‘increased’. Other authors have tried to define a useful threshold based on distribution characteristics, for example by using the mean plus one s.d. in the general population (Holzner *et al*, 2003).

The method we chose was to utilise age- and gender-adjusted norms derived from a large sample of the German general population (Schwarz *et al*, 2003). The advantage of this method is that prevalence rates are automatically controlled for the effect of age and gender, two important confounders in the area of fatigue. It is interesting to note, for example that, while the mean values of the MFI instrument are not different between the age groups, the prevalence rates using the age- and sex-adjusted norms are.

Using this approach, we determined the overall prevalence rates of 32% at the beginning of inpatient treatment, 40% at discharge, and 34% half a year after diagnosis. Cella *et al* (2001), who used diagnostic criteria instead of a dimensional instrument, determined a prevalence rate of cancer-related fatigue of 17% in patients who completed chemotherapy treatment, most of whom had breast cancer, based on meeting at least 2 of the 14 suggested criteria.

Caution should be taken when interpreting our prevalence rates longitudinally as the patient groups responding differed between t1, t2, and t3. Only when data from patients with complete data sets are used can the course of fatigue be interpreted truly longitudinally. However, the analysis of ‘completers’ alone bears potential risks too. Previous studies have shown that patients who drop out from a trial or a field study are more often distressed and have a decreased quality of life compared with the ‘completers’ (Houborg *et al*, 2006). Investigation of completers alone would have introduced a bias, leading to an underestimation of fatigue prevalence.

The analysis of different subgroups of patients, albeit with smaller numbers of study participants, gives useful hints for the planning of future trials and for clinical routine. For example, although fatigue levels generally increase with age (Schwarz *et al*, 2003), younger patients in our study had higher prevalence rates compared with older patients. Possible explanations for this are that younger patients receive more aggressive treatments, resulting in increased fatigue or, alternatively, that younger patients perceive a larger discrepancy between themselves and their peers in the general population or between their current level of energy and their previous situation.

Again, care should be taken in interpreting the subgroup prevalence rates. These analyses were not performed to assess predictors of fatigue. For example, the increased prevalence in patients with pancreatic carcinoma half a year after admittance to the hospital does not imply that the tumour site itself ‘causes’ the fatigue. It is most likely that different treatments or tumour stages have a role here as well. It has been shown, in a large longitudinal study of patients with chronic lymphatic leukaemia, that the only predictive variable for persistent fatigue was fatigue at the end of treatment (Heutte *et al*, 2009). Another study in patients with gynaecological cancer identified 12 months psychological distress as the only relevant correlate of fatigue during anticancer treatment (Prue *et al*, 2010), which is in essence a similar result. However, although predictor analysis is interesting, the knowledge of prevalence rates in different subgroups is of high clinical importance too. Therefore, we chose to present these data here.

A limitation of our study is clearly that we were not able to collect blood samples or biological data from the patients. Therefore, we cannot stratify the study group according to potential predictors such as low serum haemoglobin or albumin level, which might have been interesting.

Second, our approach to base prevalence rates on a 75th percentile is disputable. Patients could suffer from fatigue on a lower level too or, in contrast, the 90th percentile could have been more appropriate as we would be able to identify patients with severe fatigue more easily. Although fatigue was assessed by the patients themselves, thus including the amount of suffering implicitly, the impact of fatigue on daily life was not explicitly evaluated.

Another shortcoming is that the MFI, in our view, is not an optimal instrument to assess cancer-related fatigue, at least in the German translation. For some patients, some of the items are a little awkward and difficult to understand. However, other instruments have flaws too; therefore, we decided to use an instrument that is internationally known and validated. In addition, we wanted to use a multidimensional measure; therefore, the fatigue subscales of the EORTC QLQ-C30 or the FACIT were not an alternative. Our decision to use a multidimensional instrument was based on the idea that cancer-related fatigue can have an impact on daily life in several regards including not only physical but also mental and motivational aspects. At the time of the data collection for this study, the MFI was the best option for an internationally validated multidimensional fatigue instrument.

Another limitation is that we could not assess all of the patients referred to the clinic but only those who were eligible and consented to take part. Fortunately, we were able to collect the reasons for decline in most of the cases. As the two main reasons given for non-consent may be strongly fatigue-related, the prevalence rates in our study should be interpreted as conservative estimates. In other words, our study is more likely to have underestimated the true prevalence of fatigue than to have overestimated it.

In conclusion, the fatigue prevalence rates provided by this study can be used for planning future research and clinical routine. Knowing the prevalence rates in specific groups of patients is a prerequisite, for example, for planning a clinical trial assessing the effect of physical activity on fatigue, as we first need to know the ‘baseline rate’ of fatigue in that group of patients (to plan the sample size etc.).

Based on our results, we can conclude that at the beginning of cancer treatment, a third of the patients are classified as fatigued. Similar rates were observed in a recent study by Goedendorp *et al* (2008), where between 14% and 28% of the patients experienced severe fatigue before initiation of treatment. At discharge, this rate was slightly increased in our study, and half a year later, a third of the patients still report fatigue; prevalence varies significantly between different patient groups. We and others (Fayers, 2001) believe that this approach – to translate patients’ scores into their percentile position – is more easily interpretable than citing mean values.

Taking note of fatigue rates is of relevance for researchers and clinicians alike. For example, treatment schedules should be altered or made more flexible to accommodate the problems of fatigue. Support services to encourage motivation for and compliance with treatment during periods of increased fatigue could be another useful option. The age- and gender-adjusted cutoff values presented here could be used for screening purposes in clinical routine too. However, as pointed out earlier (Fayers, 2001), population norms of fatigue and other quality of life data are not necessarily comparable between countries; therefore, it is recommended that cutoff points be defined per country.

## Figures and Tables

**Table 1:** Cutoff scores used for age- and sex-standardised prevalence rates of fatigue

**Age group**	**⩽39 years**	**40–59 years**	**⩾60 years**
Men	⩽8	⩽10	⩽13
Women	⩽10	⩽11	⩽13

Note: Cutoff scores were set such that 75% of the general population in the same age and gender groups would be classified as not fatigued (Schwarz *et al*, 2003).

**Table 2:** Sample characteristics (*N*=1494)

	***N***	**%**
*Age*		
⩽39 years	96	6.4
40–59 years	508	34
⩾60 years	890	59.6
		
*Tumour site*		
Breast	166	11.1
Ovaries	38	2.5
Cervix	93	6.2
Endometrium	36	2.4
Vulva	12	0.8
Other pelvic	8	0.5
Prostate	280	18.7
Bladder	62	4.1
Kidneys	66	4.4
Testicles	29	1.9
Penis	4	0.3
Lungs	53	3.5
Oesophagus	34	2.3
Stomach	28	1.9
Pancreas	39	2.6
Liver	50	3.3
Gallbladder	24	1.6
Small intestine	7	0.5
Colon	63	4.2
Rectum	107	7.2
Malignant melanoma	26	1.7
Thyroid	14	0.9
Tongue	13	0.9
Tonsils	15	1.0
Oral cavity	12	0.8
Pharynx	45	3.0
Larynx	15	1.0
Brain	70	4.7
Eye	3	0.2
Sarcoma	8	0.5
Haematological	49	3.3
Cancer of unknown primary	8	0.5
Other	17	1.1
		
*Tumour stage (UICC)*		
0	24	1.6
I	220	14.7
II	290	19.4
III	257	17.2
IV	141	9.4
Unknown	562	37.6
		
*Tumour progression*		
Primary cancer	1144	76.6
Recurrent disease	88	5.9
Metastases	167	11.2
Secondary tumour	49	3.3
Other	30	2.0
Unknown	16	1.1

Abbreviation: UICC=Union Internationale Contre le Cancer.

**Table 3:** Fatigue at different points in time

	**t1 Beginning of inpatient treatment**		**t2 Discharge from hospital**		**t3 ½Year after baseline**		**General population**	
	**(*n*=1494)**		**(*n*=884)**		**(*n*=1035)**		**(*n*=2037)**	
	**M**	**s.d.**	**M**	**s.d.**	**M**	**s.d.**	**M**	**s.d.**
General fatigue	10.3	4.2	10.9	4.2	10.8	4.3	8.7	3.6
Physical fatigue	11.2	4.6	12.4	4.4	10.9	4.3	8.4	4.1
Reduced activity	11.4	4.7	12.8	4.6	10.9	4.6	8.4	3.8
Reduced motivation	8.8	3.8	8.8	3.9	8.9	3.8	8.0	3.3
Mental fatigue	8.8	4.1	8.9	4.3	8.6	4.0	7.7	3.3

Abbreviations: M=mean; MFI=Multidimensional Fatigue Inventory.

Displayed are the scores on each of the MFI subscales (range 4–20).

**Table 4:**
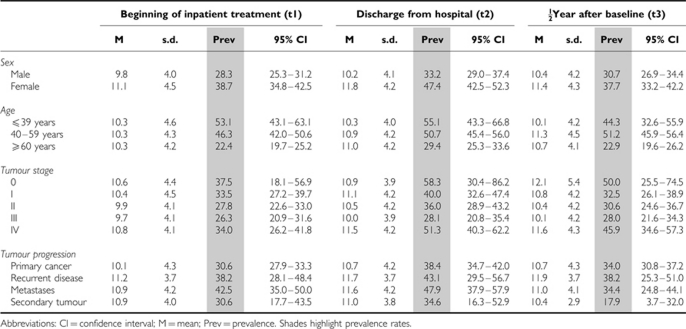
Fatigue scores and sex+age-standardised prevalence rates with 95% CI stratified by tumour stage, sex, and age

**Table 5:**
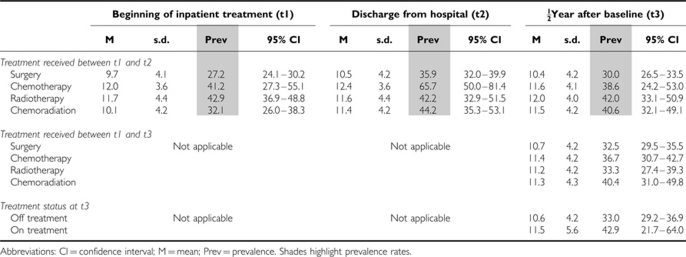
Fatigue scores and sex+age-standardised prevalence rates with 95% CI stratified by treatment

**Table 6:**
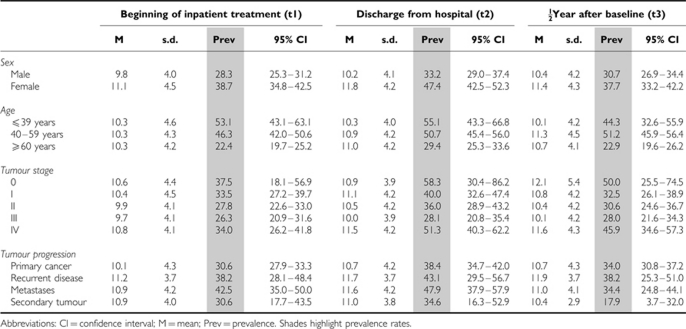
Fatigue scores and sex+age-standardised prevalence rates with 95% CI stratified by tumour site
